# Spatially coupled catalytic ignition of CO oxidation on Pt: mesoscopic *versus* nano-scale

**DOI:** 10.1016/j.ultramic.2015.05.012

**Published:** 2015-12

**Authors:** C. Spiel, D. Vogel, R. Schlögl, G. Rupprechter, Y. Suchorski

**Affiliations:** a Institute of Materials Chemistry, Vienna University of Technology, 1060 Vienna, Austria; b Department of Inorganic Chemistry, Fritz-Haber-Institute of the Max-Planck-Society, 14195 Berlin, Germany

**Keywords:** Catalytic reactions, CO oxidation, Field ion microscopy, Photoemission electron microscopy

## Abstract

Spatial coupling during catalytic ignition of CO oxidation on μm-sized Pt(*hkl*) domains of a polycrystalline Pt foil has been studied *in situ* by PEEM (photoemission electron microscopy) in the 10^−5^ mbar pressure range. The same reaction has been examined under similar conditions by FIM (field ion microscopy) on nm-sized Pt(*hkl*) facets of a Pt nanotip. Proper orthogonal decomposition (POD) of the digitized FIM images has been employed to analyze spatiotemporal dynamics of catalytic ignition. The results show the essential role of the sample size and of the morphology of the domain (facet) boundary in the spatial coupling in CO oxidation.

## Highlights

• Catalytic ignition in CO oxidation was studied on μm-sized Pt(*hkl*) domains of a Pt foil and on nanofacets of a Pt tip.
• Proper orthogonal decomposition of the FIM images has been employed to analyze dynamics of catalytic ignition.
• The effect of the sample size and morphology in the spatial coupling in CO oxidation was observed.

## 1 Introduction

Automobile engines emit a large amount of pollutants just after starting (the so called “cold-start problem”), i.e. before the catalyst in the catalytic converter reaches the “light off” temperature when the effectivity of the converter switches rapidly from a low to a high level (catalytic ignition). This effect, observed in exothermic catalytic reactions, is usually considered as a heat balance problem, with the critical ignition temperature being defined as a point where the reaction-generated heat exceeds heat dissipation [1]. In case of CO oxidation, catalytic ignition is an environmentally related topic: in order to fulfill today's stringent emission standards, sophisticated catalyst heating processes have recently been developed to quickly reach the critical temperature. These processes vary from lean air-to-fuel ratio operation, exhaust system combustion devices, secondary air injection into the exhaust, to electrically heated catalysts, etc. [2].

Alternatively, a reduced critical temperature may shorten the period between the engine start and reaction “light off”. To reveal the role of the critical temperature one has to consider that factually, catalytic ignition represents not solely a heat production problem, but is rather a convolution of reaction kinetics and heat generation, since the latter is determined by the reaction rate. The reaction rate results in turn directly from reaction kinetics. In case of model studies under high-vacuum (~10^−6^ to 10^−4^ mbar) conditions, where heat and mass transport do not play an observable role, catalytic ignition can be treated as pure kinetic phenomenon, *i.e.* as a kinetic transition from low-rate steady state to a high-rate steady state [3], [4]. Such transitions in the CO oxidation reaction are well studied, however mainly under isothermal conditions by varying the CO/O_2_ ratio [5], [6], [7]. Only few studies, *e.g.* for Pd [4], [8] or for a Pt wire [9], were performed at increasing temperature under isobaric conditions, *i.e.* modeling the “cold-start” process. Moreover, no studies have been performed to our knowledge where different length scale systems are compared with respect to isobaric kinetic transitions under the same experimental conditions.

Recently, we have developed an experimental approach where kinetic phase transitions in CO oxidation can be studied *in situ* in a spatially resolved way, *e.g.* on individual differently oriented grains of polycrystalline metal foil by analysis of local PEEM intensities [10]. The idea of this approach is based upon the fact that the local PEEM intensity (i.e. the photoemission yield) is directly dependent (via the local work function) on local CO or oxygen coverage. In turn, the rate of CO_2_ formation depends directly on CO or oxygen coverage [11]. From these two findings the dependence of the local PEEM image intensity on the local reaction rate may be concluded, which allows a spatially resolved monitoring of kinetic phase transitions [10], [12], [13].

In the present contribution we extend the above approach to the nanoscale, exploiting the fact that the local intensity of the FIM image obtained by O_2_^+^ ions depends on the oxygen coverage of the Pt-tip surface [14], [15]. In this way, catalytically active and inactive steady states of the catalyst surface can be distinguished and kinetic transitions can be monitored, as was demonstrated in our previous *isothermal* studies [16]. Herein we show the first FIM observations of the *isobaric* kinetic transitions, *i.e.* catalytic ignition of CO oxidation on the apex of a Pt-nanotip. Such an apex exhibits a heterogeneous surface formed by differently oriented nanofacets and can thus serve as a suitable model for a catalytic particle of comparable dimensions [17], [18].

As a contribution to bridging the structural complexity gap between single crystal studies and more sophisticated model systems, it would be interesting to compare the catalytic behavior of individual μm-sized grains of a polycrystalline Pt foil (as monitored by PEEM on a mesoscopic scale) with that of identically oriented facets of a Pt tip (as visualized by FIM/FEM on the nanoscale). Below we present such a comparison between [100]-, [110]- and [111]-oriented grains and identically oriented facets of a Pt nanotip focusing mainly on spatial correlation in catalytic ignition of CO oxidation for differently oriented grains (facets). We use proper orthogonal decomposition (POD, also known as a Karhunen–Loeve decomposition) of our FIM video-data to prove the synchronization of catalytic ignition on different facets of the Pt nanotip.

## 2 Experimental

The experiments were performed in two different all-metal UHV setups, as described in detail elsewhere: (i) a PEEM/XPS setup consisting of two individual PEEM and XPS chambers connected with each other by a sample transfer line [19], (ii) a FIM setup that can be operated either in the traditional FIM mode using Ne as the imaging gas (for tip preparation) or oxygen, which serves as reactant and imaging gas at the same time (for *in situ* field ion imaging of the CO oxidation reaction) [20].

The PEEM/XPS setup is equipped with a PEEM (Staib Instruments), a deuterium discharge UV lamp (photon energy ~6.5 eV) for electron excitation, an MS (MKS Instruments), XPS-system (Phoibos 100 hemispherical energy analyzer and XR 50 twin anode X-ray source, SPECS), a high purity gas supply system (O_2_: 99.999%, CO: 99.97%) and sample preparation facilities for cleaning the sample by argon ion sputtering and subsequent annealing. Differential pumping of the PEEM intensifier section by a separate turbomolecular pump and two in-line apertures along the photoelectron trajectory (diameters 4 mm and 0.3 mm) allow to keep the pressure inside the PEEM below 10^−7^ mbar, with the local pressure of reactants at the sample up to 10^−4^ mbar.

The PEEM chamber is used as a flow reactor for CO oxidation on polycrystalline Pt foil, the PEEM images were recorded *in situ* by a high-speed CCD camera (Hamamatsu). Magnification was calibrated by comparison with optical micrographs of the same Pt foil. A PEEM image, formed by photoelectrons, represents the lateral distribution of the local work function across the sample. This allows for differentiation between differently oriented grains and between different adsorbates by correlation of local image intensities with the known work function values of the corresponding clean and adsorbate-covered single crystals. The recorded PEEM video-files were related to MS-data obtained simultaneously by a quadrupole mass spectrometer (MKS) placed in the vicinity of the sample.

**Fig. 1:**
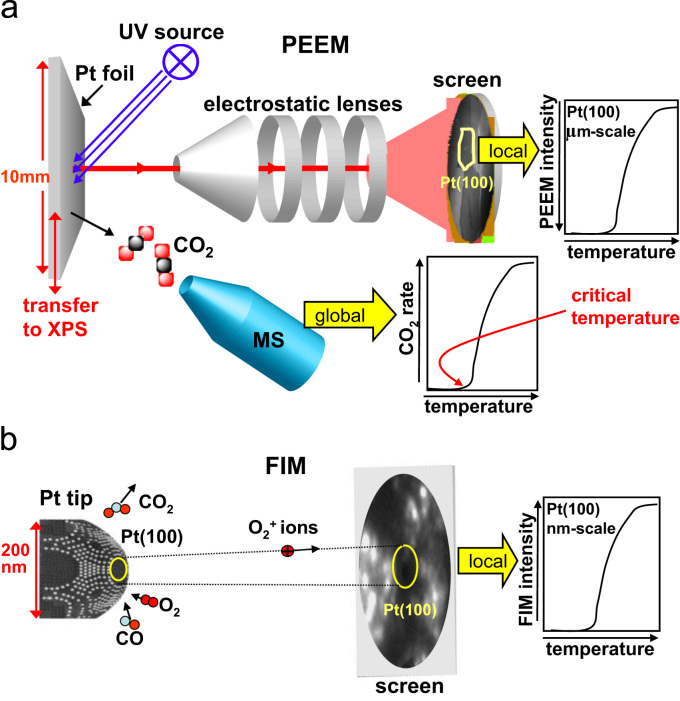
Scheme of the experiment. (a) Mesoscopic scale (Pt foil, PEEM): in a PEEM, a magnified image of the sample surface is created by photoelectrons emitted upon UV illumination, local work function generates the contrast, digital analysis of the PEEM video-data provides *local* kinetic information from individual grains of the polycrystalline foil. Simultaneous MS measurements provide the *global* reaction rate (CO_2_ formation) and (b) nanoscale (Pt nanotip, FIM): in an FIM, the image is formed by field ions of imaging gas as a “point projection”, whereas the magnification is determined by the curvature of the tip apex. Oxygen is used both as a reactant and as an imaging gas (O_2_^+^ ions), the contrast originates from the local work function and surface roughness (local field). Local image intensity provides information about the local kinetics on a nm-scale.

The PEEM sample consisted of a 10×12 mm^2^ polished polycrystalline Pt foil of 0.2 mm thickness (Mateck, 99,99%) which was flame annealed in air and further cleaned in UHV by repeated cycles of sputtering with Ar^+^ ions at 1 keV at 300 K and consecutive annealing to 973–1073 K for 30 min. The cleanness of the sample was XPS controlled after each single measurement. The sample temperature was measured by a Ni/NiCr thermocouple spot-welded to the back side of the sample. The general configuration of the experimental setup and the scheme of the PEEM experiment are shown in Fig. 1a.

In a similar way, the FIM chamber (Fig. 1b) was used as a flow reactor for CO oxidation on a Pt nanotip, the corresponding UHV system contains a tip assembly, which allows operation in a controlled temperature range of 78–900 K*,* a channel plate, a gas-supply system for imaging both noble (Ne) and reactive (O_2_, CO) gases. Additionally to the conventional DC high-voltage supply a pulsed high-voltage supply with pulses of triangular shape (with varying length (>150 ns), magnitude up to 5 kV, repetition frequencies up to 100 kHz) could be used in order to reveal the role of the applied field, as discussed below. The Pt nanotip was cleaned by field evaporation at 77 K, and the temperature of the tip was measured by a Ni/NiCr thermocouple spot-welded to its shank. FIM images during the ongoing CO oxidation reaction were recorded with the same camera as in the PEEM experiments. Fig. 1b illustrates the nanoscale (FIM) experiments.

## 3 Results and discussion

### 3.1 PEEM studies

In the usual parameter range of an automotive converter for CO oxidation, the catalytic converter system may exhibit two stable steady states, namely, a state of low reactivity with a predominantly CO covered surface (cold start), and a high reactivity state with a mostly oxygen-covered surface (optimal operating regime). Varying the external parameters, kinetic transitions between these two states can be enforced, where a hysteresis is always observed, if an external control parameter (e.g., CO partial pressure or temperature) is varied back and forth. This characteristic behavior is present also for model systems under high vacuum conditions as a manifestation of the intrinsic bistability of the catalytic CO oxidation [6], [7], [21]. Such behavior results from the Langmuir–Hinshelwood reaction mechanism due to asymmetric inhibition of the dissociative oxygen adsorption by CO. Oxygen needs two adsorption sites per molecule and can thus hardly adsorb on a densely CO-covered (poisoned) surface, whereas CO, in turn, can easily adsorb on a surface precovered with oxygen. Therefore, the recovery of a CO poisoned surface to the active state occurs at lower CO pressure than what was necessary to poison the surface – a hysteresis is observed. This means that one of two stable states of the system, with high and low reactivity, can be achieved at the same external parameters depending on the prehistory: this is an attribute of bistability.

**Fig. 2:**
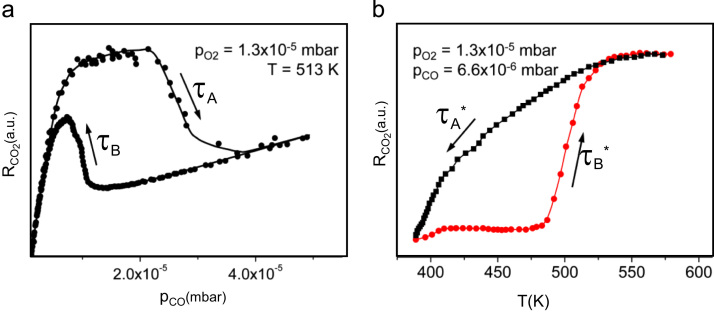
(a) Global hysteresis in the *R*_CO2_ (CO_2_ production rate, as measured by MS) for the polycrystalline Pt foil upon *p*_CO_ variation at constant *p*_O2_=1.3×10^−5^ mbar and *T*=513 K; (b) global hysteresis consisting of the ignition (red) and extinction (black) *R*_CO2_ curves upon cyclic temperature variation at constant *p*_CO_=6.6×10^−6^ mbar and at the same oxygen pressure as in (a). (For interpretation of the references to color in this figure legend, the reader is referred to the web version of this article.)

Fig. 2a illustrates such behavior in the particular case of polycrystalline Pt foil: a hysteresis in CO_2_ production is observed at cyclic varying of the CO pressure at constant oxygen pressure of 1.3×10^−5^ mbar and temperature of 513 K: transition τ*_A_* from the catalytically active (oxygen covered) to the inactive (CO poisoned) state and the reverse transition τ*_B_* take place at clearly different CO pressures. The hysteresis curve appears to be smoothened out in comparison with the known single crystal measurements [6], [7], especially the transition τ*_A_*. Such smooth appearance of the transitions τ*_A_* and τ*_B_* upon CO pressure variation reflects, as discussed in detail below, the fact that not all the grains are poisoned simultaneously, but the process occurs sequentially.

As already mentioned in Section 1, kinetic transition from the CO poisoned state to the active state can also be induced by simple temperature increase at isobaric conditions: initial desorption of CO provides more and more free adsorption places for dissociative adsorption of oxygen, this leads to an avalanche-like increase of the oxygen coverage and thus of the CO_2_ production rate. Such an isobaric kinetic transition leads under real conditions to increasing heat production, and the reaction eventually becomes self-sustained (light-off) [4], [8].

Fig. 2b shows the global ignition behavior of the polycrystalline Pt foil as monitored by MS at constant *p*_O2_=1.3×10^−5^ mbar and *p*_CO_=6.6 ×10^−6^ mbar: following the temperature ramp, the global CO_2_ production suddenly increases, the isobaric transition point τ_B_* from the low activity state to the high activity state in high-vacuum conditions corresponds (like in the present case) to the ignition point. Correspondingly, the reverse transition to the low activity state τ_A_* corresponds to the extinction point. The global extinction curve also appears to be smoothened out, and a distinct “global extinction temperature” can hardly be assigned. As will be shown below, this “smoothing” is caused by the absence of synchronization of kinetic transitions for differently oriented Pt(*hkl*) domains.

**Fig. 3:**
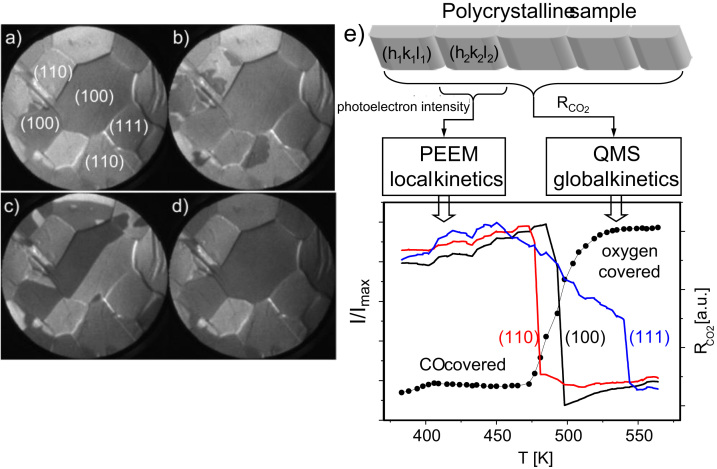
(a)–(d) PEEM video-sequence during ignition τ*_B_*⁎ on Pt foil at constant *p*_CO_=6.6×10^−6^ mbar and *p*_O2_=1.3×10^−5^ mbar. The temperature ramp has a heating rate of ~0.5 K/s from 483 K in frame (a) (CO-covered), to 492 K and 506 K in frames (b) and (c) (ignition on (110) and (100) domains) and to 568 K in frame (d) (oxygen covered). The orientation of the individual Pt(*hkl*) domains is indicated in frame (a). (e) Laterally resolved ignition/extinction measurements: local PEEM intensity for the individual (110), (100) and (111) domains during the same cyclic temperature scan as in Fig. 2b. For comparison a global curve from Fig. 2b is shown.

Monitoring the reaction evolution by PEEM provides the possibility of spatially resolved measurements. Fig. 3a–d shows a sequence of PEEM video-images taken during CO oxidation on the Pt foil at constant *p*_CO_=6.6×10^−6^ mbar and *p*_O2_=1.3×10^−5^ mbar while the temperature is ramped from 483 K (frame a) to 568 K (frame d). From local PEEM intensities read out from video-data, spatially-resolved ignition data can be extracted by analyzing local PEEM intensities from individual Pt(*hkl*) domains (Fig. 3e). In analogy to the MS signal in the overall CO_2_ reaction rate, jumps in the local PEEM intensity represent local kinetic transitions on individual grains. Since the inactive (CO covered) surface exhibits the bright contrast (low work function), the ignition jumps occur from high to low intensity and correspond to the jumps from low to high local CO_2_ production rate.

As is clearly visible from Fig. 3e, local transitions do not occur simultaneously for the different orientations but show a pronounced structure sensitivity with clearly identified critical temperatures of 417 K for Pt(110), 423 K for Pt(100), and 432 K for Pt(111). That means that individual grains “light-off” sequentially: first the [110]-oriented domains, then the [100]-oriented and then the [111]-domains. This explains immediately the smoothed character of the global curves in Fig. 2 and emphasizes the main drawback of conventional global measurements, namely, that the measured data are averaged over the whole sample consisting typically of differently active regions.

The present experiments show that individual grains of the polycrystalline Pt foil behave independent in catalytic ignition, similar as observed earlier for kinetic transitions induced by CO pressure variations [10], [12], [13]. This conclusion agrees with the observation that propagating reaction fronts are confined within grain boundaries, again analogous with the observation during cyclic CO pressure variations [12], [13]. Such independent behavior of individual Pt(*hkl*) domains allows asserting the validity of present light-off data for single crystals with corresponding orientation: e.g. the kinetic phase diagram for the Pt(111) domain on the foil (measured using the PEEM approach) coincides well with that of a Pt(111) single crystal as measured by MS [10].

### 3.2 FIM studies

Similarly as in PEEM, the contrast in FIM also depends on the local work function. However, the contrast mechanism in FIM is much more complex: whereas in PEEM the work function directly governs the photoelectron yield [22], [23], in FIM the work function influences the critical distance of field ionization and thus the probability of O_2_^+^ ion formation [24]. In addition, the atomic scale roughness of the surface modifies the local electric field [25] and thus the rate of ion formation. Last but not the least, a resonance field ionization of oxygen also contributes to the image contrast [15], but in sum, the CO covered areas appear always darker than the oxygen covered regions, at least on the platinum metal surface. For a Pt surface, this means that a FIM image appears as a “negative” of a PEEM image, but the active and inactive state in the CO oxidation can be still discriminated reliably. O_2_^+^ ions were first used for *in situ* visualization of CO oxidation already in 1990s in order to study the oscillating regime of this reaction [26]. In this work we apply this imaging mode to visualize the catalytic ignition for the first time.

**Fig. 4:**
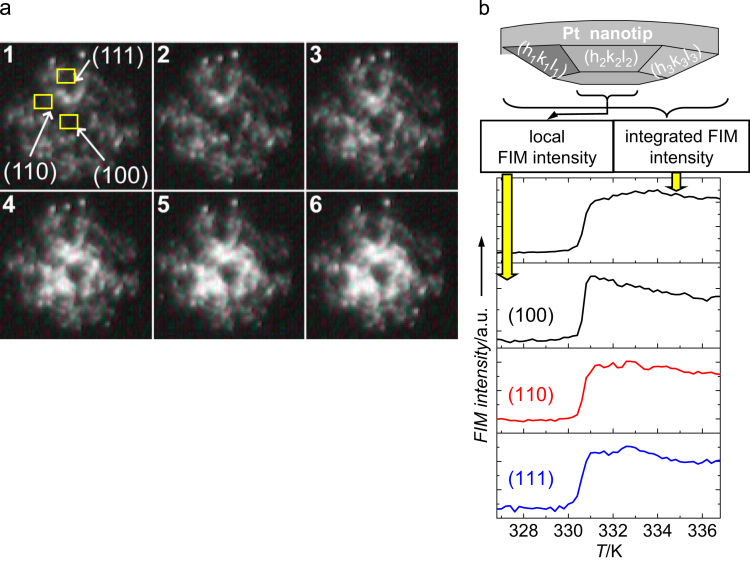
Catalytic ignition by FIM: (a) Sequence of six consecutive FIM images during catalytic ignition on a [100]-oriented Pt tip. The positions of the low Miller-index domains and corresponding regions of interests (ROIs) are shown in the first frame. Whereas in the first three frames the surface of the field emitter tip is still CO-covered and thus inactive, frames 4–6 show the catalytically active state immediately after ignition. The O_2_^+^ FIM images were recorded at a temperature of 331 K and partial pressures of CO and oxygen of 5.3×10^−7^ mbar and 5.3×10^−4^ mbar, respectively. Frame interval 0.04 s/frame.

Fig. 4a shows a sequence of six consecutive FIM images of a [100]-oriented Pt field emitter tip illustrating the transition from low to high activity upon linear increase of the temperature. The crystallography of the sample is indicated by the positions of the low Miller-index domains (frame 1 in Fig. 4a). In the first three frames of Fig.4a the surface of the Pt nanotip is still covered with CO and thus in the inactive state with a correspondingly low FIM image brightness. Between frame 3 and 4 ignition occurs: the surface gets quickly covered with oxygen, visible as a sudden increase in brightness.

Kinetic transition starts apparently from the center of the tip and instantaneously (that is, within the time span of two consecutive frames) spreads to other parts of the surface in a concentric manner.

Fig. 4b shows the corresponding FIM intensity analysis, where the integrated intensity of the whole image as well as the local intensities within the defined ROIs indicated in the first frame of Fig. 4a are shown. After a sudden rise of the FIM brightness (light-off) the intensity and size of the bright area on the sample continue to vary slightly in time. This can be attributed to fluctuations which are known from experimental studies performed on similar samples [27], [28].

Both visual inspection and local intensity analysis within the ROIs placed on low-index planes (Fig. 4b) create the impression that ignition occurs in a spatially coherent way over the majority of the facets. To obtain a profound knowledge about the existence of the coherent modes we apply local intensity analysis and proper orthogonal decomposition (POD) to the FIM video-data. POD analysis proved to be effective in the detection of coherent spatiotemporal modes, *e.g.* in hydrodynamics [29], [30]. On catalytic surfaces POD has been employed by Graham et al. to analyze spatio-temporal temperature pattern [31]. We have applied this method earlier for the analysis of reaction-induced fluctuations [32] or as a proof of the spatial desynchronization of glycolytic waves [33].

The idea of POD is based on the fact that a signal *w(**x**,t)* that varies in space and time can be decomposed into time-dependent amplitudes *a*_*n*_*(t)* and time-independent modes *b*_*n*_*(**x**)* which form an orthogonal basis:

(1) $$w\left(x,t\right)=\sum a_{n}\left(t\right)b_{n}\left(x\right)$$

**Fig. 5:**
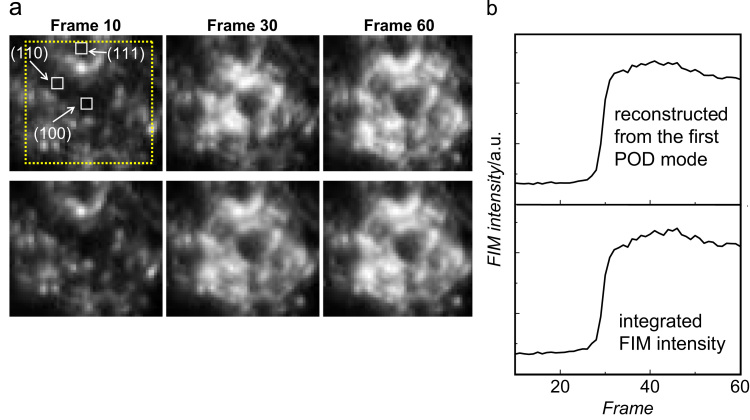
POD analysis: (a) Upper row: chosen examples of the original FIM video-frames. The ROI for the POD analysis is indicated as a dotted square and positions of the ROIs for individual orientations (the same as in Fig. 4a) are indicated. Lower row: corresponding frames calculated from the first POD mode solely (b) Upper curve: FIM intensity reconstructed from the first POD mode which contains 86% of the total signal. Lower curve: original FIM intensity integrated within the POD–ROI. The first POD mode represents well the spatio-temporal evolution.

The basis functions *b*_*n*_*(**x**)* are the *eigenvectors* of the equation:

(2) $$S\cdot b_{n}\left(x\right)=\Lambda_{n}\cdot b_{n}\left(x\right),$$

where *Λ*_*n*_ are the *eigenvalues* and ***S*** is the correlation matrix. Each eigenvalue *Λ*_*n*_ denotes the weight of the corresponding eigenvector *b*_*n*_*(**x**)*, *i.e.*, the variance of the original data set projected along this eigenvector. The eigenvectors are sorted in order of decreasing eigenvalue. The time-dependent amplitudes can be obtained by projection of the original video frames on the KL-basis *b*_*n*_*(**x**)*. If the first few eigenvectors have already most of the weight, *i.e.* they capture the overall dynamics of a system properly, the dimensionality (that is, the number of eigenvectors) of the KL-basis can be chosen much smaller than in the original without losing important information. In the present case, we use this property to obtain the main features of a complex dataset like FIM video-data. We applied POD analysis to the same FIM video-sequence that was used for the local FIM image intensity analysis (Fig. 4b). The obtained results are summarized in Fig. 5, where Fig. 5a presents a set of the original FIM video-frames (upper row) in comparison with the first POD mode (lower row).

The ROI that was chosen for the POD analysis and which includes all three low Miller-index domains (100), (110) and (111) is indicated in the first frame. Fig. 5b shows the evolution of the original FIM intensity integrated within the POD–ROI in comparison with the reconstruction performed using only the first POD mode. As can be seen from Fig. 5a,b, all main features of the intensity plots (integrated within the big POD–ROI in Fig. 5 or within the small ROIs for individual orientations in Fig. 4) are sufficiently reproduced by the first POD mode which is characterized by a sharp jump in FIM intensity at the point of the kinetic phase transition at about frame 29 and a small subsequent drop to a slightly lower level.

The image intensity then fluctuates around this level for the rest of the image sequence. Such a coincidence means that the first eigenvector, which amounts 86% of the total contribution, dominates the whole decomposition.

The higher modes contribute only little to the overall dynamics of the system, indicating a high degree of spatial correlation. Since the first POD mode represents a *constant* spatial picture which varies in accordance with Eq. (1) in time as *a*_1_(*t*), such a high contribution of the first mode means that the differently regions of the tip surface (i.e. differently oriented domains) are synchronized, i.e. the kinetic phase transition occurs simultaneously for all surface orientations. This result is in strong contrast to the observations made by PEEM for the micrometer-sized domains where a quasi-independent behavior of the individual domains was observed. The observed differences between PEEM and FIM observations shed light on the coupling mechanism in CO oxidation: since the degree of thermal coupling and of the local pressure variations (which could cause coupling through the gas phase) are similar for the Pt foil and the Pt-tip, only differences in the diffusion coupling via surface CO supply may be responsible for the observed effects. In fact, the diffusion length for CO reaches μm-range under the present reaction conditions (10^−5^ pressure range, *T*>300 K), [34], [35]. The facet size (nm-range) on a Pt tip is significantly smaller than the diffusion length of CO in the present temperature range, thus diffusive coupling provides synchronous ignition of the reaction on all the facets in the field of view. In principle, the diffusion length of CO may even provide sufficient coupling for different µm-sized domains of the polycrystalline Pt-foil surface. However, CO diffusion between the domains of a polycrystalline foil is effectively hindered by grain boundaries which confine therefore the propagation of reaction fronts within the individual domains and prevent thus the reactive spatial coupling between neighboring grains, at least under the present vacuum conditions. This suggestion is strongly supported by our recent experiments, in which a polycrystalline Pd foil was intensively sputtered by Ar^+^ ions [36]. Such sputtering fills up the cracks between the grains by Pd, enabling CO diffusion across the boundaries and, as a result, the independency of the individual (*hkl*)-domains vanishes.

## 4 Conclusions

*In situ* visualization of catalytic ignition of CO oxidation on Pt has been realized on two different length-scales: (i) using PEEM for individual grains of a polycrystalline Pt-foil (μm-scale) and (ii) by FIM for an apex of a Pt-nanotip (nm-scale). The results allow to shed light to the peculiarities of spatial coupling in this reaction: whereas the ignition process occurs independently on individual Pt(*hkl*)-domains of the polycrystalline Pt sample, the differently oriented facets of the Pt-nanotip light-off in a synchronized way. To reveal the degree of spatial synchronization, proper orthogonal decomposition (POD) was applied to the FIM video-files parallel to local intensity analysis of the FIM images. The first POD-mode contains 86% of the total contribution, confirming thus the high degree of spatial correlation on the Pt-nanotip. The obtained results can be traced back to differences in the CO diffusion paths on the polycrystalline Pt-foil and on the Pt-nanotip surface: whereas the individual facets on the tip-apex change over from one crystallographic orientation to another via stepped “transition regions” consisting solely on Pt-atoms, the individual grains on the Pt-foil surface are separated by grain boundaries consisting of cracks filled with impurities. These cracks hinder effectively CO diffusion and thus the coupling between different grains, whereas on the Pt-nanotip CO can easily diffuse from one facet to another and synchronize the catalytic ignition.
